# A Cautionary Tale: Unveiling Valentino’s Syndrome

**DOI:** 10.7759/cureus.22667

**Published:** 2022-02-27

**Authors:** Parag S Mahajan, Hatem Abdulmajeed, Abdulmalek Aljafari, Jouhar J Kolleri, Salahaldeen A Dawdi, Hussain Mohammed

**Affiliations:** 1 Clinical Imaging, Hamad Medical Corporation, Doha, QAT; 2 Medical School, Saint James School of Medicine, Arnos Vale, VCT; 3 Surgery, Hamad Medical Corporation, Doha, QAT

**Keywords:** peritonitis, gastro-intestinal perforation, acute appendicitis, peptic ulcer perforation, valentino's syndrome

## Abstract

Introduction: In the emergency room, acute pain in the abdomen is one of the most common symptoms that patients present with, and it is a result of a myriad of causes, leading to an exhaustive differential diagnosis. A perforated peptic ulcer is a rare cause of acute right iliac fossa or lower quadrant abdominal pain. It causes leakage of gastrointestinal contents in the area, resulting in localized inflammation and pain that is clinically similar to acute appendicitis. This condition is known as Valentino’s syndrome.

Aim: This study aims to highlight clinical and radiological features for patients with Valentino’s syndrome, improving diagnostic accuracy.

Methods: The authors conducted a retrospective analysis of all diagnosed cases of Valentino’s syndrome from multiple facilities within the same organization for the research study. A total of 14 nonsequential cases were gathered. The term “Valentino’s syndrome” was used to search in the PubMed and Google Scholar databases for the review of literature, and only 17 cases were found and reviewed.

Results: Of the 31 patients, 83.9% were male, with a mean age of 39 years. Of all patients who presented with abdominal pain, 25.8% had it in the lower right abdomen. Vomiting (38.7%), nausea (35.4%), fever (16.1%), and constipation were all associated symptoms (12.9%). All cases were clinically diagnosed as acute appendicitis. Many patients had elevated levels of white blood cells, neutrophils, and CRP. Computed tomography (CT) scan was used in 70.9% of the cases, followed by ultrasound (58%) and x-ray (45.1%), where pneumoperitoneum and duodenal perforations were common. Graham’s patch was used in 48.3% of the cases, appendectomy was used in 16.1% of the cases, and conservative care was used in 19.3% of the cases. Most patients were given proton pump inhibitors and antibiotics for Helicobacter pylori.

Conclusion: Timely diagnosis of Valentino’s syndrome via CT imaging is critical because it leads to immediate perforation repair. Patients’ mortality and morbidity may be reduced if they are aware of the condition and receive an accurate, rapid preoperative diagnosis.

## Introduction

Acute abdominal pain is one of the most common presenting symptoms in an emergency department [1]. Physicians consider many differential diagnoses based on the abdominal quadrant involved, ranging from self-limiting conditions to surgical emergencies [2]. Furthermore, because of the risk of genitourinary pathologies in females, the conditions considered for right and left lower quadrant pain differ in males and females. When approaching a case of acute abdominal pain, the primary goal is to distinguish the acute surgical abdomen cases from those that can be conservatively managed [3]. To do so, physicians request recommended laboratory and imaging studies based on the patient’s age and gender, location of pain, and clinical examination findings.

Right lower quadrant pain, like pain in the other quadrants of the abdomen, can be caused by a number of factors. One of the most common causes is acute appendicitis, which is caused by a nonspecific purulent infection and causes inflammation of the appendix, a blind-ending narrow tubular structure attached to the cecum [4,5]. Acute appendicitis is a medical emergency that necessitates immediate surgical intervention [6,7]. Other diseases that could mimic acute appendicitis are ureteric colic, diverticulitis, rupture of a diverticulum, mucocele of the appendix, perforated cholecystitis, pancreatitis, or colitis [8-11]. In women, the conditions could also include ovarian torsion, ruptured ectopic pregnancy, endometriosis, infarcted uterine leiomyoma, and pelvic inflammatory disease [12].

Valentino’s syndrome is characterized by a perforated duodenal, gastric, or peptic ulcer that mimics acute appendicitis. It was named after Rudolph Valentino, a famous actor who died of pleurisy after having his appendix removed. At autopsy, he was discovered to have a perforated gastric ulcer. In 2005, Hsu et al. reported a perforated duodenal ulcer in a pregnant woman that presented clinically as acute appendicitis [13]. In the English language literature search, only 17 cases of Valentino’s syndrome were found to be reported. This is the first study on Valentino’s syndrome that we know of, along with its up-to-date review of the literature.

Peptic ulcer disease (PUD) is caused by a breach in the mucosa of the stomach, the duodenum, or sometimes the lower esophagus [14]. A duodenal ulcer usually presents with symptoms of waking at night with upper abdominal pain and upper abdominal pain that improves with eating [15]. Hemorrhage, perforation, and gastric outlet obstruction are some of the complications associated with PUD [16]. The bacteria Helicobacter pylori, nonsteroidal anti-inflammatory drugs (NSAIDs), tobacco smoking, stress from other serious health conditions, Behcet’s disease, Zollinger-Ellison syndrome, Crohn’s disease, and liver cirrhosis are all potential causes of PUD [17]. The first two are the most frequently encountered etiological factors [18,19].

## Materials and methods

Aim

Here we report 14 cases of Valentino’s syndrome from multiple facilities of the same organization and review 17 cases of Valentino’s syndrome published in the English language literature till the date of writing of this manuscript. This study aims to review clinical and radiological features and treatment options for this rare condition. We are also looking for common features between our cases and the existing cases in the literature to generate a hypothesis that can be further analyzed in follow-up studies.

Methods

The authors obtained institutional regulatory board and institutional ethics committee approval from the Medical Research Center (MRC) of Hamad Medical Corporation vide project approval letter MRC-04-22-009. This is a retrospective study where the cases collected were nonconsecutive. Inclusion criteria used in the study were clinical diagnosis of acute appendicitis and imaging diagnosis of hollow viscus perforation with an absence of acute appendicitis. Cases, where acute appendicitis was not considered as the first/primary clinical diagnosis in the differentials, were excluded from the study. Also, cases, where adequate imaging data were not available, were excluded from the study. We looked through the institutional radiology information system for CT scan reports from January 2011 to November 2021 with the findings or diagnosis of “perforated OR perforation AND duodenal OR gastric OR peptic.” To avoid missing required reports due to spelling errors, multiple spelling variations of these terms were also attempted. The clinical details provided by the referring doctors were included in these reports, so reports of suspected cases of duodenal, gastric, or peptic perforation were included in the search results. These terms appeared in approximately 11,500 reports. Three radiologists manually reviewed these reports and identified 128 with computed tomography (CT) diagnoses of duodenal, gastric, or peptic perforation. A more thorough search was conducted within the referral details of these reports to identify cases with the primary clinical diagnosis/suspicion of acute appendicitis. This study included a total of 14 cases that were discovered. The term “Valentino’s syndrome” was searched for in the PubMed and Google Scholar databases for the review of literature, and the 17 cases found were reviewed.

## Results

A total of 14 cases of Valentino’s syndrome were discovered in our organization’s medical records (Table 1), in addition to 17 cases discovered online through a literature review (Table 2), for a total of 31 cases. Males made up 26 (83.9%) of the total number of patients. One of the five females was pregnant. The ages of those who presented ranged from seven to 76 years, with a mean of 39 years.

**Table 1:** Analysis of data from patients with Valentino’s syndrome, within the authors’ organization M = male, F = female, NA = not applicable, UA = upper abdomen, RLQ = right lower quadrant, PU = periumbilical, Epi = epigastric, V = vomiting, N = nausea, C = constipation, B/l = bilateral, GP = Garaham’s patch, hrs = hours, D1 = first part of the duodenum, d = days

No.	Age	Sex	Pregnant	Site of abdominal pain	Symptom	H/O NSAIDS intake	WBC count/microliter	Neutrophil %	CRP level mg/L	Serum amylase level U/L	X-ray	US	CT	Appendix position	Management	Time gap	Site of perforation in surgery/CT	Postop, compl.	Hospital stay
1	32	M	NA	UA to RLQ	V	No	13,700	76	< 5	nil	No pneumop	Not done	pneumop	Paracecal	Diagnostic Lap	18 hrs	D1	No	5 d
2	36	M	NA	PU to RLQ	V	No	10,600	73	nil	23	Not done	Not done	pneumop	Paracecal	Laparotomy	20 hrs	pylorus	No	2 d
3	39	M	NA	Diffuse	nil	No	13,800	83	337	50	Not done	Free fluid	pneumop	Subcecal	Diagnostic Lap	7 hrs	D1	No	7 d
4	49	F	No	Diffuse	N, V	Yes	9,900	74	21	29	No pneumop	Free fluid in abd	pneumop	Retrocecal	Lap repair, GP	13 hrs	D1	No	6 d
5	24	M	NA	UA to RLQ	N	No	19,300	87	< 5	nil	Not done	B/ll pleural effusion	pneumop	Retrocecal	Lapa repair, GP	12 hrs	D1	No	9 d
6	41	F	No	PU to RLQ	nil	No	6,500	71	nil	22	Not done	unremarkable	pneumop	Retrocecal	Lap repair, GP	25 hrs	pylorus	No	6 d
7	23	M	NA	Epi to RLQ	N, C	No	12,300	85	< 5	nil	Not done	Not done	pneumop	Paracecal	Diagnostic lap	7 hrs	pylorus	No	7 d
8	26	M	NA	Epi to RLQ	V, C	No	13,600	82	nil	49	pneumop	no free fluid	pneumop	Retrocecal	Conservative	NA	D1	NA	6 d
9	46	M	NA	RLQ	N	Yes	14,500	74	72	nil	pneumop	Free fluid in abd	No pneumop	Paracolic	Conservative	NA	D1 by CT	NA	10 d
10	53	M	NA	PU to RLQ	N	No	10,600	68	182	60	Not done	Free fluid in abd	pneumop	Paracolic	Diagnostic lap	26 hrs	D1	No	7 d
11	43	M	NA	RLQ	nil	No	11,700	67	121	19	No pneumop	Not done	pneumop	Paracolic	Conservative	NA	D1 by CT	NA	3 d
12	38	M	NA	RUQ to RLQ	nil	No	20,000	88	83	nil	No pneumop	Not done	pneumop	Paracolic	Conservative	NA	D1 by CT	NA	8 d
13	36	M	NA	RUQ to RLQ	V	No	12,600	82	343	15	No pneumop	unremarkable	No pneumop	Retrocecal	Conservative	NA	D1 by CT	NA	9 d
14	27	M	NA	Diffuse	N	No	17,600	94	113	38	Not done	Not done	pneumop	Retrocecal	Lap repair, GP	13 hrs	D1	No	4 d

**Table 2:** Analysis of data from patients identified through literature review M = male, F = female, NA = not applicable, RLQ = right lower quadrant, PU = periumbilical, LLQ = left lower quadrant, LA = lower abdomen, Epi = epigastric, Hypo = hypogastric, F = fever, V = vomiting, N = nausea, C = constipation, AUD = air under the diaphragm, GP = Graham’s patch, D1 = first part of the duodenum, D1, D2, D3 = first, second, and third parts of the duodenum, PPI = proton pump inhibitor, H. pylori = Helicobacter pylori

No.	Name of article	Age	Sex	Pregnant	Site of abdominal pain	Sympt	H/O NSAID intake	WBC/µL	X-ray	US	CT	Management	Site of perforation	Postop complications	Stay in hospital postop	Follow-up
1	Sgro [20]	32	M	Na	RLQ	F	No	16,900	No	Free fluid in RIF	Not done	Lap appendectomy, GP	Not available	None	Not available	Healed D. ulcer
2	Wijegoonewardane [21]	30	F	No	RLQ	-	No	13,300	No	Free fluid pelvis	Not done	Lap appendectomy	Not available	None	3 days	Healed ulcer
3	Yildiz [22]	17	M	NA	Not available	-		21,100	Normal	Free fluid in RIF	Not done	Laparotomy, GP	Stomach	None	5 days	None
4	Mohan [23]	17	M	NA	RLQ	F, V	No	Elevated	AUD	Free fluid pelvis	No pneumop	Laparotomy, GP	D1	None	Not available	PPI for 15 days
5	Munoz Abraham 1 [24]	16	M	NA	PU to RLQ		Yes	23,000	No	Not done	Pneumop	Diag. laparoscop, GP	D1	None	5 days	PPI, anti H. pylori
6	Munoz Abraham 2 [24]	16	M	NA	LLQ	N	Yes	Nil	No	Not done	Pneumop	Diag. laparoscopy, GP	Stomach - gr. Curvature	None	5 days	PPI, anti H. pylori
7	Mbarushimana [25]	12	M	NA	LA	V	No	19,600	AUD	Not done	Not done	Diag. laparoscopy, GP	D1	None	6 days	Anti H. pylori
8	Iloh [26]	45	M	NA	epi to RLQ	V, F	No	15,900	Not done	Free fluid in abd	Not done	Emergency laparotomy	Stomach - peripyloric	Surgical superficial site infection	3 weeks	Anti-H. pylori
9	Mahajan [27]	21	M	NA	LA & epi	F, V	Yes	23,000	Pneumop	Not done	Pneumop	Diag. laparoscopy	D1	none	6 days	Normal
10	Luna Guerrero [28]	52	M	NA	PU to RLQ	N, V		Nil	Not done	Free fluid in abd	Not done	Explo. Laparotomy with GP	Not available	-	-	-
11	Hussain [29]	7	M	NA	Epi to RLQ pain	None	No	12,700	Not done	Not done	Not done	Open appendicectomy, GP	D1	None	Not available	Healed D. ulcer
12	Noussios [30]	51	M	NA	RLQ	N, V	No	17,700	Normal	Not done	Not done	Explo. Laparotomy with simple closure	Duodenum	Not applicable	9 days	PPI, anti H. pylori
13	Amann [31]	18	F	No	RLQ	N, V	Yes	6,200	Not done	Not done	Pneumop	Explo. Laparoscopy, GP	D1	None	3 days	Anti H. pylori
14	Hsu [13]	23	F	Yes	Epi to RLQ	None	No	11,900	Not done	Free fluid in abd	Not done	Appendectomy	D1 - bulb	None	12 days	None
15	Chavez 1 [32]	26	M	NA	Hypo to RLQ	N, V	Yes	14,500	Normal	Free fluid in abd	Pneumop	Diag. laparoscopy, GP	Stomach - antrum	None	Not available	Not available
16	Chavez 2 [32]	76	M	NA	PU to RLQ	F, N, C	No	12,500	Normal	Free fluid	Not done	Lap. Appendectomy, GP	D2	None	7 days	Not available
17	Wang [33]	72	M	NA	RLQ	-	Yes	Nil	Pneumop	Retropneumop	Pneumop	Conservative	D2, D3	None	Not applicable	Not available

The presenting complaint about all patients was abdominal pain, eight (25.8%) of which had predominantly right lower abdominal pain, six (19.3%) had predominantly epigastric pain, and the other six had predominantly periumbilical pain. Other presentations included diffuse abdominal pain (three patients [9.7%]) and predominantly hypogastric pain (one patient with hypogastric pain radiating to right lower quadrant). Sixteen (51.6%) patients had their pain radiating to the right lower quadrant from another site. Associated symptoms were most commonly vomiting (38.7%), nausea (35.4%), fever (16.1%), and constipation (12.9%). Acute appendicitis was the most frequently diagnosed clinical condition. NSAID use was reported by eight (25.8%) of the patients. WBC counts were available for 26 patients, with the mean at a presentation being 14,550/µL. The mean percentage of total WBC count at a presentation of neutrophils for cases found in our organization was 78.85%; however, the neutrophil count was not available for cases found through a literature search. Eight patients had elevated CRP levels, with a mean at a presentation of 158.9 mg/L, three had normal levels (10 mg/L), and the remaining patients had no information.

The imaging modalities most often used were CT (70.9%), followed by ultrasound (58%) and x-ray (45.1%), respectively. The most common findings were pneumoperitoneum and duodenal perforations. Fluid collections in the pelvis and abdomen, including the right iliac fossa, right perinephric region, subhepatic region, Morrison’s space, pericholecystic region, right paracolic region, and cul-de-sac, were also discovered using CT and ultrasound. On CT, fat could be seen standing around the duodenum at times. Only cases found in our institution had information regarding appendix position, the most common presentation of which was retrocecal (42.8%), followed by paracolic (28.5%) and paracecal (21.4%). Out of the total sample, 27 (87%) patients had a known site of perforation. The first part of the duodenum and the stomach were the most common.

Surgical repair with Graham’s patch was used for 15 (48.3%) patients, whereas six (19.3%) patients were managed by conservative treatment. Five (16.1%) patients were treated with appendectomy. Peritonitis was seen in laparoscopy in four patients, none of which were seen in imaging. For those who underwent surgery in our institution, the time gap between clinical diagnosis and surgery was 15.6 h on average. No postoperative complications were reported, excluding one patient who had a superficial surgical site infection. The postoperative stay period ranged from two days to three weeks, with a mean of 6.72 days of stay. Proton pump inhibitors and H. pylori antibiotics were prescribed for most patients.

Case 1

**Figure 1:**
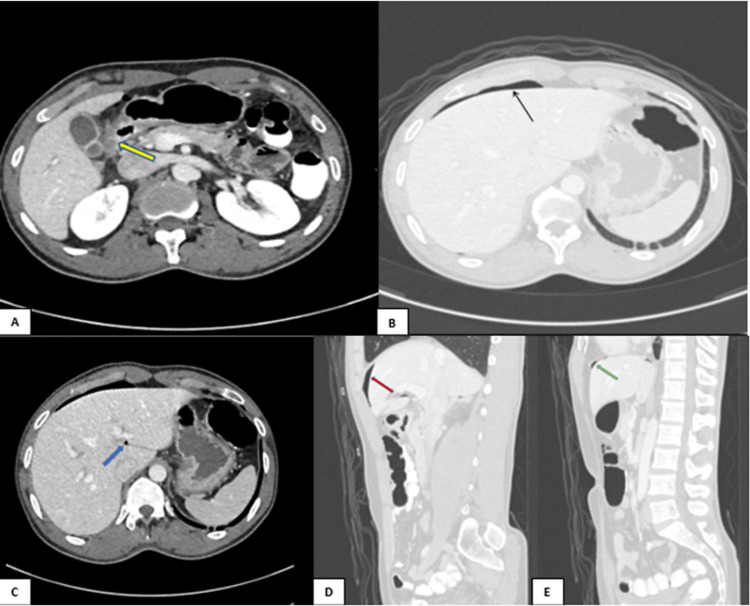
CT abdomen with peroral and IV contrast (A-C) axial and (D, E) sagittal reformatted images showing mural thickening, irregularity, and abnormal wall enhancement involving the pyloric region and the first part of the duodenum (yellow arrow) with a trace of pericholecystic fluid and fat stranding surrounding the antrum and proximal duodenum. There is intraabdominal free air along the porta hepatis (blue arrow), anteriorly in the upper abdomen (red arrow), and in the subdiaphragmatic region (green arrow).

A 32-year-old man was brought to the emergency department complaining of pain in his right lower quadrant. To rule out acute appendicitis, a CT scan of the abdomen with peroral and intravenous (IV) contrast revealed mural thickening, irregularity, and abnormal wall enhancement involving the pyloric region and the first part of the duodenum, as well as a trace of pericholecystic fluid and fat stranding surrounding the antrum and proximal duodenum (Figures 1A-1E). The presence of intraabdominal free air was observed along the porta hepatis, anteriorly in the upper abdomen, and in the subdiaphragmatic region. Valentino’s syndrome was suggested by these findings.

Case 2

**Figure 2:**
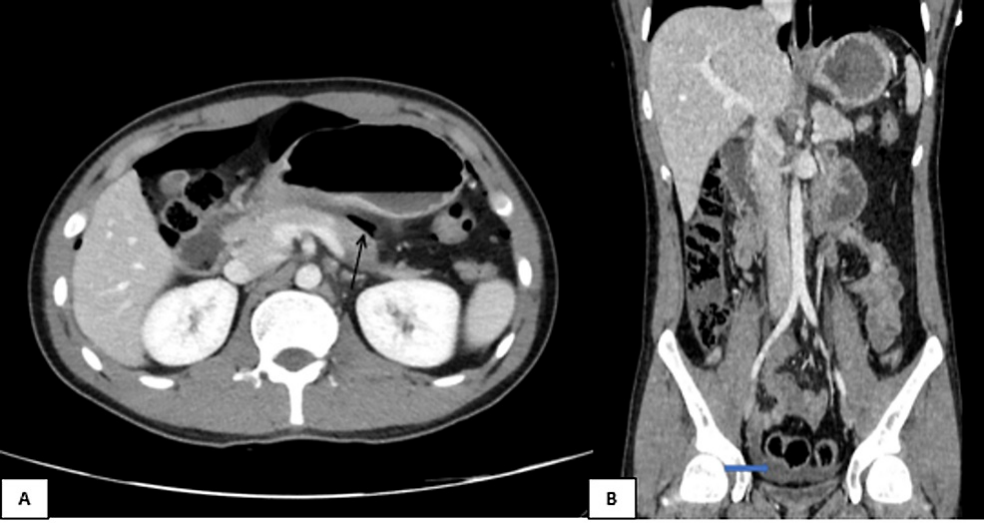
CT abdomen with IV contrast (A) axial and (B) coronal reformatted images showing pneumoperitoneum with free air in the lesser sac (black arrow) and free fluid in the pelvis (blue arrow).

A 23-year-old man presented a case of right lower abdominal pain, vomiting, positive rebound tenderness, and elevated white blood cells. A CT abdomen with IV contrast was performed, revealing significant pneumoperitoneum with minimal free fluid around the stomach’s pylorus. Valentino’s syndrome was suggested by the presence of free air in the lesser sac and pelvis (Figures 2A, 2B).

Case 3

**Figure 3:**
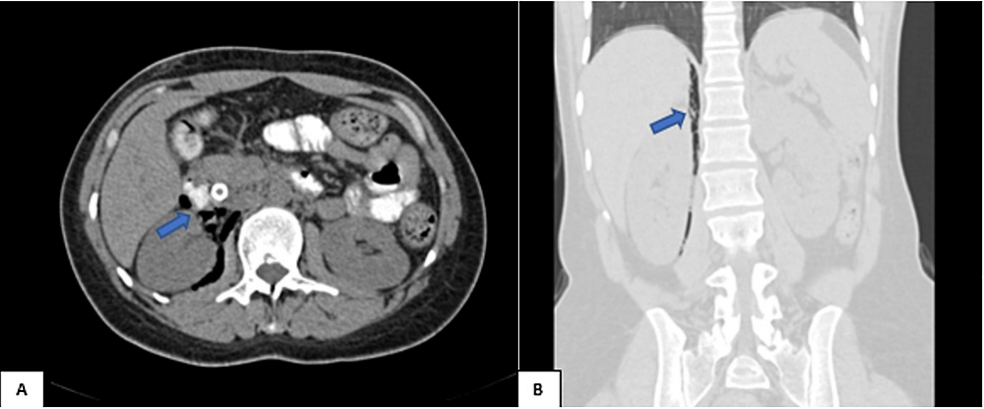
CT abdomen with IV contrast (A) axial and (B) coronal reformatted images showing perineal pocket of air masking right kidney (blue arrows).

CT abdomen with IV contrast demonstrating perirenal pocket of air masking right kidney, which is called a veiled kidney sign (Figures 3A, 3B).

## Discussion

Gastrointestinal diseases in general, including peptic ulcers, have a heavy economic and social burden on our society. PUDs cost the United States an estimate of $3.1 billion annually [34]. Peptic ulcers are caused by a combination of defensive factors (e.g., mucus-bicarbonate layer and prostaglandins) that protect the mucosa and aggravating factors (e.g., hydrochloric acid) that cause mucosa necrosis. Peptic ulcers can be caused by a number of factors, the most common of which is the overuse of NSAIDs, which inhibit COX and prostaglandin synthesis [35]. Furthermore, stress, irregular or unhealthy eating habits, H. pylori infections, and rare cases such as Crohn’s disease, Zollinger Elision syndrome, and Cushing’s syndrome or complications of malignancy, chemotherapy, or radiotherapy can all lead to peptic ulcer formation [36].

When gastric or duodenal fluid leaks from an ulcer and accumulates in the right paracolic gutter, it causes peritonitis that mimics acute appendicitis and results in Valentino’s syndrome [13,27,37]. Pneumoperitoneum is frequently caused by intraperitoneal perforation of the first part of the duodenum, whereas pneumo-retroperitoneum is caused by retroperitoneal perforation [21].

In our study, eight (25.8%) of the patients reported a history of NSAID use. This is consistent with the current evidence linking NSAID use to PUD [38]. Because of a lack of data in the cases included in our review, only two patients were reported to have a history of positive H. pylori infection. A CT scan is the imaging modality of choice for diagnosing Valentino’s syndrome. In our study, it was also the most commonly used modality in diagnosing cases (70.9%). Ultrasound and x-ray were also frequently used. The ability of a CT scan to detect small amounts of extraluminal gas distinguishes it from other imaging modalities in diagnosing retroperitoneal duodenal perforations [21].

Pneumoperitoneum and duodenal perforations were common findings in our research. On CT scanning, retroperitoneal air around the right kidney and the region of the duodenum, as well as duodenal thickening, are considered characteristic findings [6]. The air around the kidney, also known as veiled kidney signs, can be seen on abdominal radiographs [39]. CT findings also included occasional fat standing around the duodenum and fluid collections in the pelvis and the abdomen in areas such as the right perinephric region and the right iliac fossa. In our study during a laparoscopy, four patients were discovered to have peritonitis, none of which was visible on imaging. Usually, patients undergo an operation for an acute surgical abdomen with suspicion of acute appendicitis, but when the appendix is found to be normal without inflammation, the intraoperative investigation leads to the diagnosis of a perforated peptic ulcer [33].

The required treatment for Valentino’s syndrome is a surgical fixation of the perforated duodenal ulcer. It can be performed via open laparotomy or laparoscopy. Surgical repair with Graham’s patch was the most common method of treatment (48.3%), although conservative management was also used with six (16.1%) patients. In patients suspected to have peptic ulcers, NSAIDs should be avoided to prevent complications like perforation. In the case of a perforated gastric ulcer, an intraoperative biopsy should be performed to exclude malignancy [38].

The use of triple-drug therapy for the elimination of H. pylori is mandatory in proven cases of gastroduodenal ulcers as recommended by Prabhu et al. [36]. Conservative treatment for perforated gastroduodenal ulcers is a therapeutic alternative known as Taylor’s method. This method is reliable in a selected population and consists of Ryle’s tube aspiration, fluids resuscitation, antibiotics, and antisecretory drugs [30].

Early detection and intervention may result in lower patient mortality and morbidity, whereas delayed treatment may increase the risk of risks such as breakdown of repair, surgical site infection, and wound dehiscence, as well as an increase in hospital stay and cost of care [40].

## Conclusions

To the best of our knowledge, this is the first original article with a large number of cases and literature review of Valentino's syndrome, which is more significant considering its rare incidence. The diagnosis of Valentino's syndrome via CT imaging is easy and can help in avoiding the surgery or directing the surgeon directly to the repair of the duodenal perforation. Consequently, it is critical for emergency physicians, surgeons, and radiologists to be aware of this entity and consider it in their differential diagnosis, as its early detection may significantly reduce patient morbidity and mortality.
